# Uterine artery embolization for the treatment of symptomatic adenomyosis: a systematic review

**DOI:** 10.1186/s42155-026-00727-2

**Published:** 2026-07-15

**Authors:** Corrado Ini’, Concetta Timpanaro, Pietro Valerio Foti, Francesco Tiralongo, Renato Farina, Federica Libra, Davide Giuseppe Castiglione, Francesco Vacirca, Roberto Milazzotto, Corrado Spatola, Maria Chiara Lo Greco, Giuseppe Messina, Emanuele David, Stefano Palmucci, Antonio Basile

**Affiliations:** 1 Department of Medical Surgical Sciences and Advanced Technologies “G.F. Ingrassia” – Radiology I Unit, University Hospital Policlinico “G. Rodolico-San Marco”, Via Santa Sofia 78, Catania, 95123 Italy; 2 NANOMED-Research Centre for Nanomedicine and Pharmaceutical Nanotechnology, University of Catania, Catania, 95125 Italy; 3 Centro di Ricerca Multidisciplinare “Chirurgia delle Sindromi Malformative Complesse della Transizione e dell’Età Adulta” (ChiSMaCoTA), Department of Medical Surgical Sciences and Advanced Technologies “G.F. Ingrassia”, University of Catania, Catania, 95123 Italy; 4 Radiation Oncology Unit, University Hospital Policlinico “G. Rodolico-San Marco”, Catania, 95123 Italy; 5 Radiation Oncology Unit, Department of Biomedical, Dental and Morphological and Functional Imaging Sciences, University of Messina, Messina, 98122 Italy; 6 UOSD I.P.T.R.A., Department of Medical Surgical Sciences and Advanced Technologies “GF Ingrassia”, University of Catania, University Hospital Policlinico “G. Rodolico-San Marco”, Catania, Italy

**Keywords:** Adenomyosis, Uterine artery embolization, Radiology interventional, Abnormal menstrual bleeding, Chronic pelvic pain

## Abstract

**Background:**

Adenomyosis is a benign uterine condition characterized by the presence of glands and endometrial stroma in the myometrium, with hypertrophy of the smooth muscle cells and uterine enlargement, and causing chronic pelvic pain, dysmenorrhea, and abnormal uterine bleeding, resulting in significant impairment of quality of life. In the last decades, the role of interventional radiology has expanded the range of treatment options of this condition, and uterine artery embolization (UAE) has emerged as a minimally invasive therapy in the management of symptomatic adenomyosis. This systematic review aims to evaluate the efficacy and safety of UAE in patients with symptomatic adenomyosis.

**Methods:**

A systematic literature search was conducted across PubMed, Embase, Cochrane Library, Google Scholar, and Medline database, including studies published up to December 2025. Eligible articles included prospective and retrospective observational cohort studies with more than ten patients reporting UAE outcomes for adenomyosis; case report, case series, narrative or systematic review, meta-analysis, and guidelines were considered as not eligible and were therefore excluded. The data analyzed included patient characteristics, technical and clinical success, symptom improvement, imaging outcomes, reintervention rate, and complications, the latter classified according to the new modified CIRSE classification grading system.

**Results:**

Twenty-two studies met the inclusion criteria and we collected data on 1701 patients who underwent uterine embolization for therapy-resistant adenomyosis. UAE has demonstrated high technical success rates across studies with 1301/1701 (76.4%) patients experienced significant improvement in heavy menstrual bleeding, dysmenorrhea, and bulky-related symptoms at short- and mid-term follow-up. Imaging assessments showed reductions in uterine volume and junctional zone thickness; reintervention and hysterectomy rates were low. Reported complications were predominantly minor, with a low incidence of major adverse events. The mortality rate post-procedure was 0%.

**Conclusions:**

Based on available literature, UAE represents a safe and effective uterus-preserving minimally invasive treatment for selected patients with symptomatic adenomyosis; it represents a step on the treatment pathway before hysterectomy, and women should have access to consult with interventional radiologists when making any treatment decision, in order to achieve a personalized treatment plan.

## Introduction

Adenomyosis is a common nonneoplastic gynecologic condition characterized by the presence of ectopic endometrium within the myometrium, with hyperplastic/hypertrophic smooth muscular tissue around ectopic endometrial glands. First observed by Rokitansky in 1860 as “cystosarcoma adenoides uterinum”, and then described by Von Recklinghausen, Cullen, and Frankl, the definition of adenomyosis proposed by Bird et al. in 1972, describing adenomyosis as the benign invasion of endometrial tissue into the myometrium, resulting in a diffusely enlarged uterus with ectopic endometrial glands and stroma surrounded by hypertrophic and hyperplastic myometrium, remains widely accepted today [1–3].

### Epidemiology and risk factors

Adenomyosis typically affects multiparous women in premenopausal stage and it can manifest with dysmenorrhea and heavy menstrual bleeding. The prevalence of adenomyosis in hysterectomy specimens varies from 8 to 70%, and the disease is increasingly diagnosed in younger women, including those experiencing infertility, pelvic pain, or abnormal uterine bleeding [4]. However, epidemiologic data are fragmented due to differing diagnostic criteria (histological definition or the imaging modality used), the primary reliance on hysterectomy for diagnosis, and the absence of general population screening [5]. According to the latest studies, the development of adenomyosis is associated with risk factors, such as high estrogen levels, early age of starting menstruation, shorter menstrual cycles, higher body mass index (BMI), endometriosis, use of tamoxifen for breast cancer treatment, uterine surgeries, or genetic and epigenetic alterations. Protective factors against the condition have also been identified, although they are still subject to debate, in particular smoking and breastfeeding (associated with lower estrogen levels and reduced risk among parous women).

### Etiopathogenesis

The exact cause of adenomyosis is unknown, but different pathogenetic theories were postulated on how endometrial glands directly invade the myometrium.

The “invagination theory” suggests that adenomyosis develops when endometrial basalis cells or cell groups invade into the myometrium through an injured or abnormal junctional zone, forming ectopic adenomyotic lesions and affecting the myocytes in the inner myometrium and outer myometrium. Migration of endometrial cells into the myometrium is facilitated by enhanced invasion capacity of endometrial stromal fibroblasts, especially when exposed to myocytes from women with adenomyosis [6]. The irregular distribution of myocytes and disruptions in the nuclear membrane within the inner myometrium, associated with adenomyosis, suggest that this compartment may play a role in the disease’s development. This has led to the proposition that both physical and physiologic trauma, often referred to as “microtrauma”, to the endometrial-myometrial interface contribute to adenomyosis pathogenesis [6]. Although the endometrial invagination theory is widely supported as the main mechanism behind adenomyosis, other pathways may also be at play. For instance, adenomyosis lesions have been found in the myometrium of patients with Rokitansky-Kuster-Hauser syndrome, where functional endometrium is absent. This suggests that adenomyosis might develop through alternative mechanisms, such as metaplasia or differentiation of embryonic or adult endometrial stem cells within the myometrium. During the development and merging of Mullerian ducts, some embryonic tissue remnants might get displaced into the myometrium, potentially leading to adenomyosis later in life. These remnants could undergo metaplastic changes, giving rise to ectopic endometrial tissue [6]. Apart from the direct migration of stem cells from the endometrial basalis into the myometrium, there is another potential pathway involving retrograde menstruation. Perivascular endometrial mesenchymal stem cells (eMSCs) found in both the basalis and functionalis layers of the endometrium are present in menstrual blood as well. Chapron et al. proposed the “from outside to inside invasion” theory, suggesting that adult endometrial cells or stem cells carried in retrograde menstrual flow could infiltrate the uterine serosa and penetrate into the outer myometrium, giving rise to intramyometrial endometrial implants or adenomyotic foci. This theory finds support in the frequent coexistence of posterior focal adenomyosis and deep infiltrating endometriosis nodules in the posterior compartment among patients with endometriosis/adenomyosis [6].

### Diagnosis

The diagnosis of adenomyosis, particularly in relation to abnormal uterine bleeding (AUB), has become a significant component in the FIGO (International Federation of Gynecology and Obstetrics) PALM-COEIN classification, closely associated with excessive menstrual bleeding [7]. The gold standard for the diagnosis of adenomyosis was mainly based on histologic analysis after the removal of the uterus, looking for specific signs such as the presence of misplaced endometrial glands and a peculiar stroma at a minimum depth of 2.5 mm below the surface of the endometrium, along with changes in the surrounding muscle tissue [8]. Nowadays, the diagnosis of this condition is also possible without the use of invasive procedure. Transabdominal sonography (TAS) and transvaginal sonography (TVS) are commonly used as the initial imaging modality for patients with clinically suspected adenomyosis, with a low reported sensitivity for the former and high specificity for the latter [9, 10]. Magnetic resonance imaging (MRI) is an accurate, noninvasive modality for diagnosing adenomyosis, with a high sensitivity and specificity, identifying the location and extent of the disease, and distinguishing adenomyosis from leiomyoma and other pathological conditions owing to its exceptional tissue contrast resolution and multiparameter properties [5, 11–14]. Computed tomography (CT) has low sensitivity for adenomyosis but may suggest the diagnosis based on uterine enlargement, thickened inner myometrium, and/or myometrial cysts. CT is also useful for identifying other abdominal and pelvic conditions [15].

### Treatment

Uterine adenomyosis has a great impact on patients’ daily life, it can affect fertility and pregnancy, and it could heighten the likelihood of adverse outcomes in pregnancy and for newborns, such as preeclampsia, preterm birth, and small-for-gestational-age (SGA) patients. Treatment of adenomyosis encompasses both medical and surgical therapies. Among the first, oral contraceptives (OCs) are prescribed to alleviate menstrual bleeding by inducing decidualization and subsequent endometrial atrophy. This treatment approach can be particularly beneficial for patients experiencing dysmenorrhea and heavy menstrual bleeding, as it often leads to amenorrhea, providing relief from symptoms. Gonadotropin-releasing hormone agonists (GnRH-a), controlled by the hypothalamic–pituitary–gonadal axis, effectively ease chronic pelvic pain from adenomyosis, lessen menstrual flow, and enhance chances of conception. Commonly used GnRH-a types include leuprorelin acetate, goserelin acetate, and triptorelin. Mifepristone, an established systemic steroid, emerges as a promising therapeutic option for adenomyosis, exerting its effects through multifaceted mechanisms involving cell viability, apoptosis, migration, and uterine volume reduction [16]. Hysterectomy is currently the only definitive therapy for adenomyosis, reserving for patients with severe pain not responding to other therapies and in whom fertility is no longer desirable. In recent decades, hysterectomy has been replaced by conservative uterine-saving methods and reproductive-sparing therapies. High-intensity focused ultrasound (HIFU) employs ultrasound beams to induce coagulative necrosis in targeted adenomyotic lesions, offering a noninvasive approach; however, its suitability depends on lesion visibility, making it less effective for diffuse adenomyosis. In recent years, the role of interventional radiology in clinical practice has expanded the range of treatment options available for adenomyosis. Uterine artery embolization (UAE) allows, through a minimally invasive approach, to alleviate adenomyosis symptoms by closing off uterine blood vessels and reaching a state of hypoxia and ischemia of the ectopic endometrium, resulting in necrosis and absorption of proliferative cells and connective tissue. These events achieve the purpose of relieve or disappear of clinical symptoms [17, 18].

Even if potential utero-ovarian anastomoses can facilitate the embolization of the ovarian artery by decreasing blood supply, the impact of UAE on ovarian reserve is not significant [19]. In fact, recent studies have shown that FSH and LH levels post-UAE return to normal after 12 months, indicating a low incidence rate of ovarian dysfunction, particularly in young women, since they can exhibit a greater capacity for recovery from ovaries damaged on the long-term follow-up [20, 21]. UAE has the advantages of less trauma, rapid recovery, uterine normal physiological and reproductive preservation, and reduction of costs and length of hospitalization compared to more invasive alternative therapies. Furthermore, UAE allows the release of therapeutic agents into the target lesions, reducing systemic toxicity, complication rate, uterine volume, and relieving dysmenorrhea [22].

The purpose of our study is to analyze and review techniques, common clinical practice, outcomes, and safety of UAE for the treatment of uterine adenomyosis. This manuscript systematically reviews most recent clinical trials, prospective and retrospective studies concerning this topic.

## Materials and methods

### Systematic review

Based on the “PICOS (Population/Problem, Intervention, Comparison, Outcome, and Study design)” criteria and “Preferred Reporting Items for Systematic Review and Meta-Analysis (PRISMA)” guidelines, an extensive systematic literature search in the field of uterine artery embolization for adenomyosis was performed. The literature search included PubMed, Embase, Cochrane Library, Google Scholar, and Medline databases and the following medical subject headings (MeSH) and keywords, associated with Boolean operators, without truncation/wildcards, were used to identify main articles relevant to our purpose: “adenomyosis embolization” OR “adenomyosis UAE” AND “uterine artery embolization for adenomyosis” OR “uterine artery embolization for the treatment of adenomyosis” OR “interventional radiology.”

The “PICOS” items formed the basis of the research question and included the following information: P (patients with diagnosis of adenomyosis on imaging), I (minimally invasive treatment), C (comparison between different interventional radiology techniques), O (effectiveness outcomes, adverse effects, and adverse effects limiting effectiveness of treatment), S (retrospective study, clinical trial phase III, randomized controlled phase III trial, phase II trial reports, prospective pilot study, retrospective cohort analysis, multicentric randomized trial, other prospective studies).

### Eligibility criteria

Strict inclusion and exclusion criteria were applied. Only human studies, articles written in English, and those where the entire content was accessible were included in the present review. No interval in the literature search period was specified and the authors screened studies up to December 2025. Prospective, retrospective observational or cohort studies with more than ten patients were included, while case report, case series with fewer than ten patients, narrative or systematic review, meta-analysis, and guidelines were considered as not eligible and were therefore excluded. Articles not compatible with the aims of our research due to the use of not specific MeSH and keywords and recurring articles from the same authors on the same procedure were excluded.

### Patients

The study population inclusion criteria were as follows: adult female of childbearing age (aged 18 or older) with a diagnosis of adenomyosis on imaging; patients with failure of medical therapies; patients desiring for uterine preservation without fertility impairment. The exclusion criteria were as follows: pregnancy; patients with uterine malignant tumors; patient with pelvic inflammatory disease, autoimmune disorders, or immunosuppressed; patients without pelvic imaging; patients with an Eastern Cooperative Oncology Group (ECOG) performance status > 2.

### Intervention

In the present systematic review, only studies focusing on UAE with minimally invasive approach were included. Studies focusing on both adenomyosis and fibroid treatments were also included if only they provided specific results related to adenomyosis.

### Outcome measures

Treatment techniques and materials used were analyzed for each procedure. Whenever available, data on technical and clinical success were recorded. Technical success was achieved with stasis of contrast in the distal ascending segment of the uterine artery on both sides after embolization. The statistics on technical and clinical success, complications, and adverse events were also compared when specified. Outcome parameters evaluated were as follows: symptom improvement after UAE (chronic pelvic pain, menstrual disorders, dysmenorrhea, menometrorrhagia, dyspareunia), morphological changes post-treatment (uterine volume reduction, junctional zone changes—thickness, necrosis), reintervention rate, pregnancy rate after first UAE. Symptoms were evaluated using different quality of life questionnaires and pain scales. Complications and adverse events, with or without clinical implications, were analyzed during the follow-up period and standardized using the modified CIRSE classification grading system [23]. Adverse events and complications were also divided into minor (grade 1–2–3) and major (grade 4–5–6).

### Study selection

**Fig. 1:**
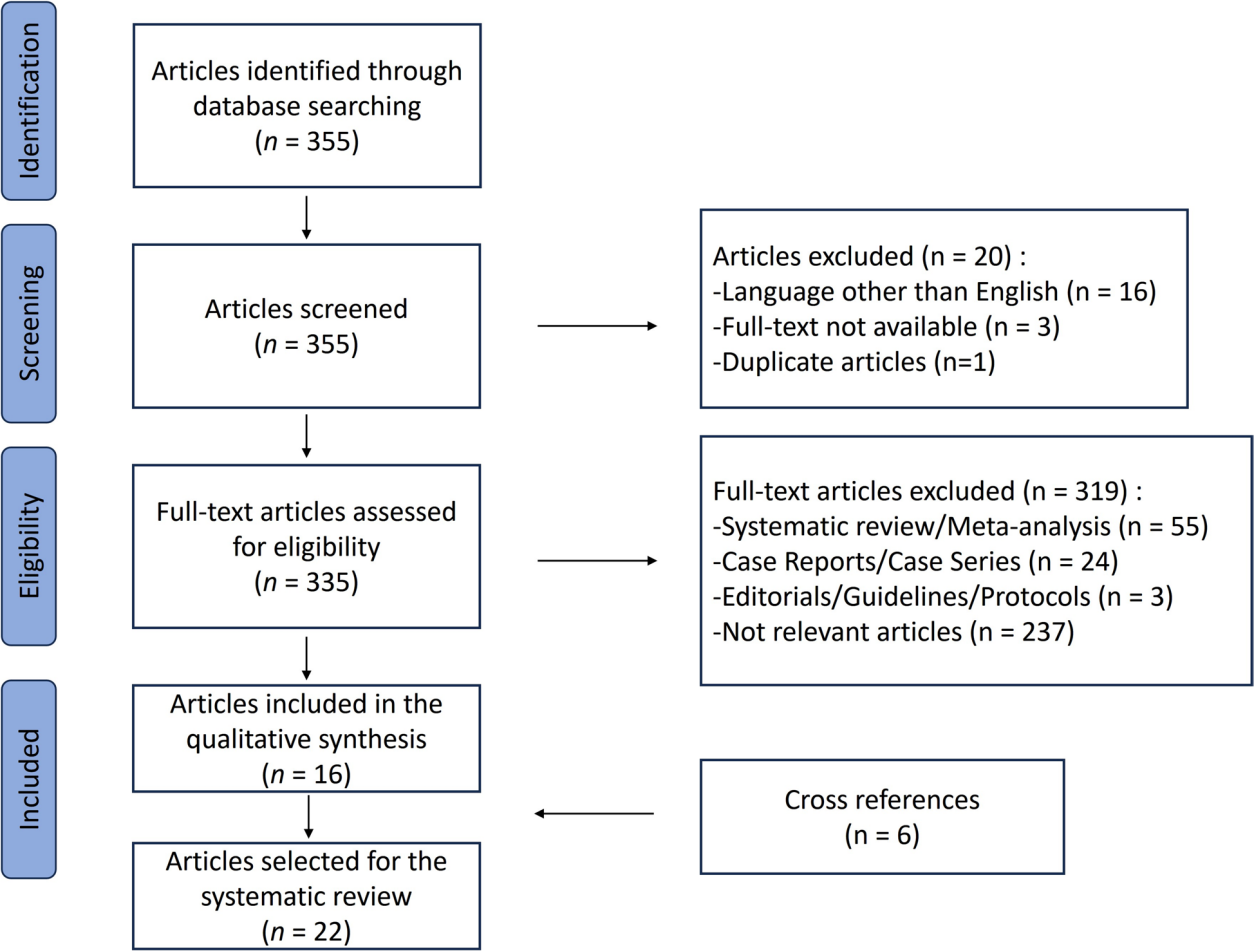
Flowchart illustrating selection process of the articles included in the systematic review

Study selection was conducted using Rayyan.ai (https://www.rayyan.ai), an online platform designed to facilitate the screening process of the articles [24]. All included articles based on inclusion and exclusion criteria, keywords, and MeSH were examined by two different reviewers among authors and disagreement over the literature data were settled through discussion among the other authors. Prioritization feature of Rayyan.ai was used solely to support the screening process by ranking studies according to their predicted relevance based on the eligibility criteria; all final screening decisions were made exclusively by human reviewers. Data were recorded using Microsoft Excel database (Microsoft Corporate, version 2403, Redmond, WA, USA), indicating first author, year of publication, design of the study, number of patients enrolled, outcomes, and complications. Based on the aforementioned search criteria, 355 records were identified and of which 20 were removed because they were duplicated or written in a language other than English or because the full-text was not available. Fifty-five articles were removed because they were systematic review or meta-analysis, and 27 articles were excluded because they were case reports or case series or editorials or guidelines. After the analysis of titles and abstract of full-text articles, 237 studies were removed as they were not relevant for the purpose of our review, since they did not meet inclusion criteria. Articles considered for our research were therefore reduced to 16 papers; further 6 articles have been added analyzing cross-references from previous included studies. The systematic review of the literature was finally conducted on a total of 22 articles [25–46]. A flowchart, illustrated in Fig. 1, summarized the whole process of selecting studies based on the inclusion and exclusion criteria.

Our study did not directly involve humans and therefore did not require the Institutional Review Board approval of our institution.

### Quality assessment and risk of bias

The Newcastle–Ottawa scale was used to assess the quality and risk of bias of non-randomized studies (case control studies and cohort studies) (Tables 1, 2) [47, 48].

**Table 1:** Risk of bias based on Newcastle–Ottawa scale

First author, year	Selection				Comparability	Outcome			Total
	Exposed representation	Selection of non-exposed cohort	Ascertainment of exposure	Outcome of interest not present at start of study		Assessment of outcome	Sufficient follow-up time	Adequacy of follow-up of cohort	
Bratby, 2009 [25]	+	−	+	−	−	+	+	−	4
De Bruijn, 2017 [26]	+	+	+	−	−	+	+	−	5
Froeling, 2012 [27]	+	−	+	−	−	+	+	−	4
Guo, 2021 [28]	+	−	+	−	−	+	+	−	4
Hu, 2024 [29]	+	−	+	−	−	+	+	−	4
Kim M.D., 2007 [30]	+	−	+	−	−	+	+	−	4
Kim M.D., 2011 [31]	+	−	+	−	+	+	+	−	5
Kitamura, 2006 [32]	+	−	+	−	−	+	+	+	5
Liang, 2018 [33]	+	−	+	−	−	+	+	−	4
Manduca, 2025 [34]	+	−	+	−	+	+	+	+	6
Mitranovici, 2025 [35]	+	+	+	−	+	+	+	+	7
Nijenhuis, 2015 [36]	+	−	+	−	−	+	+	−	4
Pelage, 2005 [37]	+	−	+	−	−	+	+	−	4
Siskin, 2001 [38]	+	−	+	−	−	+	+	−	4
Smeets, 2012 [39]	+	−	+	−	−	+	+	−	4
Trommelen, 2025 [40]	+	+	+	−	+	+	+	+	7
Turtòczki, 2024 [41]	+	−	+	−	−	+	+	−	4
Wang S., 2016 [42]	+	−	+	−	−	+	+	−	4
Wang Y., 2020 [43]	+	−	−	−	−	+	+	−	3
Wei, 2025 [44]	+	−	+	−	+	+	+	−	5
Yuan, 2021 [45]	+	−	+	−	−	+	+	−	4
Zhou, 2016 [46]	+	−	+	−	−	+	+	−	4

**Table 2:**
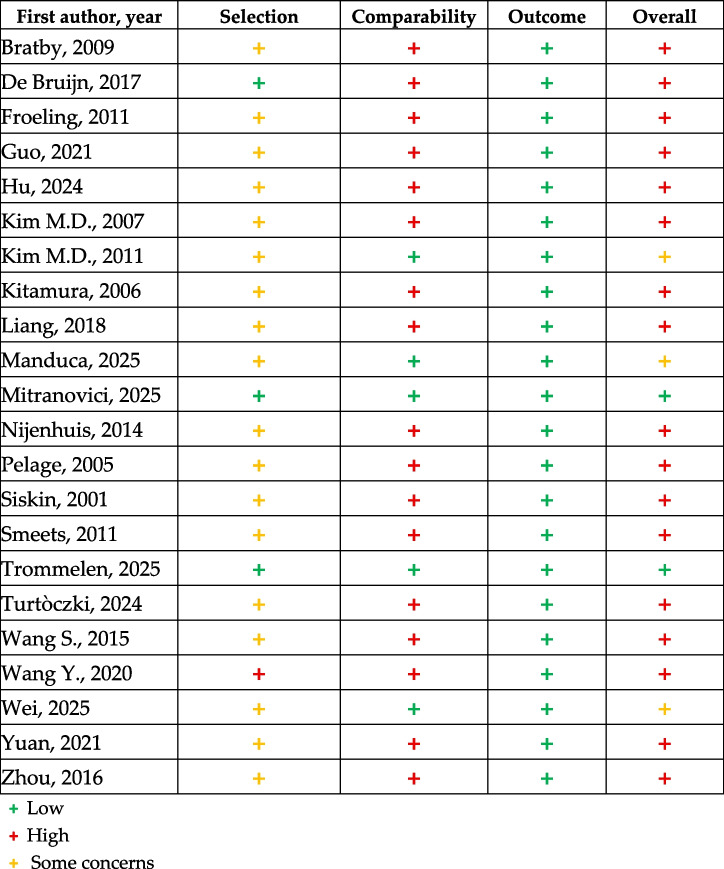
Overall risk of bias based on Newcastle-Ottawa scale [25–46]

### Definitions

In last years, minimally invasive therapies have provided a viable alternative to more invasive surgical therapies in different fields of medicine. This approach offers a safe and effective option for appropriately selected women, reducing costs and the length of hospitalization. UAE technique in adenomyosis is similar to that used in fibroids. The procedure is performed under conscious sedation, with a minimally invasive arterial vascular access, under fluoroscopic guidance, and with selective and super selective arteriography of both uterine arteries and their branches. The standard approach for UAE is via the femoral artery; in fact, femoral artery is a large vessel that is easy to approach allowing artery embolization being performed repeatedly. However, this approach could limit the patient’s quality of life and increase the risk of complications, such as deep vein thrombosis, arterial bleeding, and formation of pseudoaneurysm. The transradial artery approach has become the gold standard in coronary interventions, and promising results were also obtained in UAE. By accessing the distal radial artery, this approach reduces pain, radiation exposure, and access site complications, offering faster recovery and quicker patient mobilization rather than femoral artery approach. The most frequent complication of transradial approach is the vessel spasm after puncture and the occlusion of the radial artery in the snuffbox, which can be prevented through administration of nitroglycerin [49]. To date, further studies are needed to demonstrate the superiority of the transradial approach over the transfemoral approach for UAE of adenomyosis [50]. The embolic material is introduced into the bloodstream in a fractionated manner (free-flow embolization) once the catheter is positioned in the horizontal segment of the uterine artery, and the angiogram indicates satisfactory flow. Occasionally, spasm leads to the complete halt of flow, which should be managed with the intra-arterial administration of nitroglycerin or tolazoline [15]. Embolization is usually performed using variable-sized permanent particulate agents. Extensive experience has been documented with polyvinyl alcohol (PVA), Gelfoam, and tris-acryl gelatin-coated microspheres (TAGM). Particles of non-spherical shape, measuring 350–750 μm, and microspheres ranging in size from 500 to 900 μm are also utilized [15]. The angiographic endpoint achieved with non-spherical PVA is blood stasis, indicating complete blockage of the flow in uterine arteries [15]. Special attention is paid to visualizing the cervicovaginal and ovarian artery branches. Distal embolization avoids vaginal necrosis and the unwanted reflux of microspheres into the ovarian artery. After completing embolization on the opposite side, the uterine artery on the same side is catheterized either by creating a Waltman loop or by using a curved catheter, such as the Rösch inferior mesenteric catheter, which functions as a hook and it can be inserted into the internal iliac artery with ease [15].

The aim of UAE is to relieve symptoms, mainly pain, while preserving normal physiological and reproductive functions of the uterus. UAE induces more than 34% necrosis within adenomyotic tissues. In fact, by embolizing the uterus and focal blood supply, the ectopic endometrium enters a state of hypoxia and ischemia, resulting in necrosis dissolution and absorption of proliferative cells and connective tissue. These events lead to symptom relief and uterine morphological changes.

## Results

According to the inclusion and exclusion criteria, 22 studies were selected for inclusion in the present systematic review, encompassing a total of 1701 female patients treated with UAE for adenomyosis. The mean age of the entire population included in the study was 42.9 years (range, 27–64 years). Fifteen out of 22 articles (68.1%) were retrospective studies and 7/22 articles (31.8%) were prospective studies. Eleven out of 22 (50%) articles reported patients with pure adenomyosis and adenomyosis associated with fibroids, 1/22 (4.5%) articles reported patients with adenomyosis associated with endometriosis and 10/22 (45.4%) articles reported patients with pure adenomyosis. Six out of 22 articles (27.2%) focused on differentiating the various forms of adenomyosis, which can be focal, diffuse, symmetrical, or asymmetrical. Symmetric diffuse adenomyosis was defined as uniform widening of the junctional zone, whereas asymmetric diffuse adenomyosis was diagnosed when adenomyosis was diffuse but dominant on one side of the endometrial canal. Focal adenomyosis, or adenomyoma, was diagnosed when the region of adenomyosis was more localized and appeared as an oval poorly marginated mass-like lesion on imaging. In all studies, the main symptoms evaluated were heavy menstrual bleeding, dysmenorrhea, pelvic pain, and bulk-related symptoms (sensation of pressure on the bladder and/or rectum, abdominal swelling, urinary frequency, constipation). Heavy menstrual bleeding was the prevalent symptom among patients with an incidence more than 70%, followed by dysmenorrhea and bulky symptoms. Adenomyosis was diagnosed through cross-sectional imaging (MRI and US) in all studies and, in particular, with only US in one study, with MRI in 17 studies, and with a combination of MR and US in 4 studies. In one study, five patients underwent transvaginal biopsy to confirm the diagnosis.

The UAE technique used among studies was transcatheter selective embolization of uterine arteries, and technical success was achieved in 99.8% procedures. In 14/22 studies, a transfemoral artery approach was used to gain arterial access, while in 2/22 studies a transradial artery approach was used; in 6/22 studies, the type of arterial puncture was not specified by the authors. All procedures were conducted under local anesthesia or conscious sedation. Different types of embolic agents were analyzed in all studies. Calibrated microparticles (sizes ranging from 100 to 710 µm) have been used in all procedures. In 12/22 articles, polyvinyl alcohol particles (PVA) with a diameter ranging from 100 to 700 µm were used as the main embolic agent for UAE. In some cases, the particle size was increased during the procedure to achieve complete embolization of target vessels. In 10/22 studies, tris-acryl gelatin microspheres (TAGM) were used by authors for adenomyosis embolization, and, in two studies, polyzene F-coated hydrogel microspheres were used [26, 36]. The main characteristics of the studies included in this systematic review are summarized in Table 3.

**Table 3:** Main characteristics of the studies included in the systematic review

First author, year	Type of study design	Number of patients	Type of treatment	Embolic material (dimensions)
Bratby, 2009 [25]	Retrospective	27	UAE	PVA (355–500 µm)
De Bruijn, 2017 [26]	Prospective	29	UAE	Polyzene F-coated hydrogel microsphere (–)
Froeling, 2012 [27]	Retrospective	40	UAE	TAGM, PVA (355–900 µm)
Guo, 2021 [28]	Retrospective	76	UAE	TAGM (500–900 µm)
Hu, 2024 [29]	Retrospective	48	UAE	TAGM (500–900 µm)
Kim M.D., 2007 [30]	Retrospective	66	UAE	PVA (250–710 µm)
Kim M.D., 2011 [31]	Prospective	40	UAE	PVA (150–500 µm)
Kitamura, 2006 [32]	Prospective	19	UAE	TAGM, PVA (355–700 µm)
Liang, 2018 [33]	Retrospective	117	UAE	PVA (300–700 µm)
Manduca, 2025 [34]	Retrospective	76	UAE	Embosphere (300–700 µm)
Mitranovici, 2025 [35]	Retrospective	25	UAE	PVA (150–250 µm)
Nijenhuis, 2015 [36]	Prospective	29	UAE	Polyzene F-coated hydrogel microsphere (500–900 µm)
Pelage, 2005 [37]	Prospective	18	UAE	TAGM (355–900 µm)
Siskin, 2001 [38]	Retrospective	15	UAE	PVA (355–500 µm)
Smeets, 2012 [39]	Retrospective	40	UAE	Embosphere (500–900 µm)
Trommelen, 2025 [40]	Prospective	50	UAE	Embosphere (300–700 µm)
Turtòczki, 2024 [41]	Retrospective	15	UAE	PVA (500–700 µm)
Wang S., 2016 [42]	Prospective	115	UAE	TAGM (500–700 µm)
Wang Y., 2020 [43]	Retrospective	195	UAE	PVA (–)
Wei, 2025 [44]	Retrospective	382	UAE	Embosphere (100–700 µm)
Yuan, 2021 [45]	Retrospective	27	UAE	PVA (100–300 µm)
Zhou, 2016 [46]	Retrospective	252	UAE	PVA (355–710 µm)

*PVA* polyvinyl alcohol, *TAGM* tris-acryl gelatin microspheres

Twenty-one out of 22 articles (95.4%) reported the duration of follow-up, ranging from 3 months to 7.4 years (mean follow-up duration of 28.4 months). The follow-up was conducted through imaging (MRI, US) and/or questionnaires. Symptoms of adenomyosis were evaluated before and after treatment and their improvements (primary outcome) were tested through different scales and questionnaires (VAS, HRQOL, UFS-QOL, QoF, SSS). Abnormal menstrual bleeding was also assessed with health-related quality of life (HRQOL) questionnaire and symptom severity scores (SSS) scale in 7/22 articles (31.8%), and with Quality of Life (QoF) questionnaire in 8/22 articles (36.3%), while pelvic pain and dysmenorrhea were assessed with Visual Analogue Scale (VAS). In two study, Chronic Pain Grade questionnaire and American Fertility Society (AFS) scoring system were used as outcome assessor, and in one study the type of questionnaire was not specified. All studies (100%) reported type and rate of symptom improvement after UAE.

Symptom improvement was observed in at least 50% of the patients, reaching up to 96% in some cases. Overall, 1301/1701 patients experienced significant symptom improvement, with an average percentage of 76.4%. Most studies (> 50%) showed an improvement in symptoms in more than 70% of patients who underwent UAE. Abnormal menstrual bleeding was the main symptom improved after treatment both in a short- and long-term follow-up, followed by dysmenorrhea and bulky symptoms.

In 5/22 studies (22.7%), a secondary attempt of embolization was performed to treat persistent symptoms, and in 13/22 (59%) studies secondary hysterectomy was reported in 55/1701 patients (3.2%) after UAE for recurrence of symptoms.

Morphological changes (uterine volume, maximal junctional zone thickness) were analyzed in 16/22 studies (72.7%) and were considered as outcome parameters for the treatment. Uterine volume and junctional zone thickness significantly decreased after UAE. The uterine volume before treatment was reported in 16/22 (72.7%) studies and ranged from 236 to 790 cm^3^, with a mean uterine volume of 396.6 cm^3^, and after treatment ranged from 151 to 569 cm^3^, with a mean uterine volume of 240.3 cm^3^. The mean percentage reduction in uterine volume was 36.2%. Maximal junctional zone thickness before treatment was reported in 11/22 studies (50%) and ranged from 12 to 70 mm, with a mean value of 27.9 mm. In 8/22 studies (36.3%), authors measured junctional zone thickness after UAE, with a mean reduction of 8.7 mm (34.4%). Adenomyosis necrosis was considered as a predictor of good outcomes after UAE and was measured as an area of absence of contrast enhancement on T1-weighted images. However, this data was present only in 4/22 articles (18.8%) with a complete necrosis in 71.6% of cases of adenomyosis and partial necrosis in 68.8, 73.6, and 5% of cases in three different studies respectively. Post-operative outcomes and morphological changes available for the studies analyzed in the present systematic review are summarized in Tables 4 and 5.

**Table 4:** Mean follow-up times and clinical response following UAE

First author, number of patients	Symptom improvement, *n* (%)	Follow-up (months)	Secondary hysterectomy, *n* (%)
Bratby, 27	24 (88%)	36	2 (7%)
De Bruijn, 29	21 (74%)	88	5 (17%)
Froeling, 40	92 (72%)	40	10 (25%)
Guo, 76	59 (78%)	12	–
Hu, 48	31 (64%)	36	–
Kim M.D. 2007, 66	50 (76%)	57	5 (8%)
Kim M.D. 2011, 40	19 (47%)	14	–
Kitamura, 19	16 (84%)	12	–
Liang, 1170	102 (87%)	22	6 (5%)
Manduca, 76	71 (93%)	7	7 (9%)
Mitranovici, 25	23 (92%)	6	1 (4%)
Nijenhuis, 29	22 (76%)	37	1 (3%)
Pelage, 18	12 (67%)	24	5 (28%)
Siskin, 15	12 (80%)	8	–
Smeets, 40	29 (72%)	65	7 (17%)
Trommelen, 50	28 (56%)	13	–
Turtòczki, 15	14 (93%)	65	1 (7%)
Wang S., 115	108 (94%)	12	–
Wang Y., 195	124 (63%)	15	4 (2%)
Wei, 382	151 (39%)	3	–
Yuan, 27	26 (96%)	42	1 (4%)
Zhou, 252	134 (53%)	60	–

**Table 5:** Morphological changes before and after UAE reported among studies

First author	Uterine volume (mean) before UAE	Uterine volume (mean) after UAE	JZ thickness (mean) before UAE	JZ thickness (mean) after UAE
Bratby	528 cm^3^	–	–	–
De Bruijn	–	–	–	–
Froeling	381 cm^3^	–	31 mm	–
Guo	–	–	–	–
Hu	266 cm^3^	–	41 mm	–
Kim M.D., 2007 [30]	279 cm^3^	188 cm^3^	–	–
Kim M.D., 2011 [31]	347 cm^3^	197 cm^3^	–	–
Kitamura	439 cm^3^	293 cm^3^	36 mm	31 mm
Liang	296 cm^3^	198 cm^3^	23 mm	17 mm
Manduca	748 cm^3^	258 cm^3^	17 mm	4 mm
Mitranovici	–	–	–	–
Nijenhuis	351 cm^3^	231 cm^3^	24 mm	15 mm
Pelage	297 cm^3^	262 cm^3^	–	–
Siskin	455 cm^3^	231 cm^3^	30 mm	21 mm
Smeets	–	–	19 mm	12 mm
Trommelen	236 cm^3^	–	12 mm	–
Turtòczki	298 cm^3^	182 cm^3^	41 mm	29 mm
Wang S	790 cm^3^	485 cm^3^	–	–
Wang Y	331 cm^3^	208 cm^3^	–	–
Wei	–	–	–	–
Yuan	304 cm^3^	151 cm^3^	33 mm	24 mm
Zhou	–	–	–	–

*JZ* junctional zone, *UAE* uterine artery embolization

Post-procedure complications were divided into minor (grade 1–2–3) and major (grade 4–5–6), according to the new modified CIRSE classification system, based on additional post-procedure therapy or prolonged hospital stay, and permanent sequelae, including death. The analysis of complications was available in 15/22 studies (68.2%), while in 7/22 studies (31.8%) the type of complication was not analyzed, limiting safety conclusions. Patients experienced a minor grade of post-procedure complications and pelvic pain was the main symptoms after UAE (127/1701, 7.4%), followed by nausea and abnormal uterine/vaginal bleeding; infection (endometritis, urinary tract infections) was reported in 9/1701 patients (0.5%). In one patient, a major grade of complication occurred (uterine necrosis) and in 25/1701 patients (1.4%) uterine synechiae and intrauterine adhesions (Asherman syndrome) were associated with infertility. Permanent amenorrhea occurred in 45/1701 patients (2.6%) during follow-up, but was not perceived as a complication by most patients. No mortality rate was reported among the studies. The number, rate, and type of complications in the individual studies are summarized in Table 6.

**Table 6:** Complication rate and adverse effects after UAE

First author	Grade 1 (a-b), grade 2* [*n* (%)]	Grade 3 (a-b)* [*n* (%)]	Grade 4–5* [*n* (%)]	Grade 6* [*n* (%)]
Bratby	N/A**	N/A**	N/A**	N/A**
De Bruijn	N/A**	N/A**	N/A**	N/A**
Froeling	N/A**	N/A**	N/A**	N/A**
Guo	0	0	0	0 (0%)
Hu	N/A**	N/A**	N/A**	N/A**
Kim M.D., 2007 [30]	N/A**	N/A**	N/A**	N/A**
Kim M.D., 2011 [31]	0	0	0	0 (0%)
Kitamura	0	0	0	0 (0%)
Liang	3 (2%): groin hematoma	3 (2%): endometritis, IVU	0	0 (0%)
Manduca	8 (10%): pelvic pain, urinary retention	0	0	0 (0%)
Mitranovici	0	0	1 (4%): uterine necrosis	0 (0%)
Nijenhuis	0	2 (7%): infection	1 (3%): pseudoaneurysm	0 (0%)
Pelage	0	7 (39%): pain, vaginal discharge	0	0 (0%)
Siskin	0	15 (100%): nausea, vomiting, pain	0	0 (0%)
Smeets	0	0	0	0 (0%)
Trommelen	N/A**	N/A**	N/A**	N/A**
Turtòczki	0	0	1 (7%): amenorrhea	0 (0%)
Wang S	64 (56%): nausea	112 (97%): pelvic pain	0	0 (0%)
Wang Y	3 (1%): pain	7 (4%): abnormal menstrual bleeding, infection	25	0 (0%)
Wei	0	0	0	0 (0%)
Yuan	0	1 (4%): endometritis	4 (15%): amenorrhea	0 (0%)
Zhou	N/A**	N/A**	N/A**	N/A**

^*^Grading according to modified CIRSE classification system for complications reporting

^**^Not available

## Discussion

Clinical and imaging presentations of adenomyosis pose significant medical challenge in women. In some people, adenomyosis causes no symptoms or only mild discomfort, while for others, symptoms can be disabling, drastically worsening patients’ quality of life. Even if the exact cause of adenomyosis is unknown, its growth depends on the hormone estrogen. Endometrial glands directly invade the myometrium resulting in vessel angiogenesis, smooth muscle hyperplasia, and hypertrophy. These changes prevent uterine contractions from tamponing bleeding myometrial arterioles, leading to heavy menstrual bleeding.

Therapy of adenomyosis depends on the severity of symptoms and the need to preserve fertility. First-line therapy aims to control pain with conservative pharmacological treatments, including nonsteroidal anti-inflammatories (NSAID), hormonal contraceptive medications, or intrauterine devices; the use of a levonorgestrel intrauterine device has shown promising results in symptom relief [51]. If conservative measures fail in controlling symptoms, invasive options can be considered, with hysterectomy representing the definitive treatment choice. However, nowadays patients and physicians are more prone to opt for minimally invasive therapies. This approach has several advantages since uterus-preserving techniques allow to maintain fertility and reduce perioperative morbidity and the length of hospital stay associated with surgery. Ablative techniques, MR- or ultrasound-guided high-intensity focused ultrasound (HIFU), and transcatheter embolization represent the most recent uterine-sparing minimally invasive techniques. However, ablative therapies and HIFU are subject to certain limitations including availability, overall cost, and unknown fertility outcomes; more consolidated experience is developing for UAE. Over the past 15 years, UAE has been studied for the treatment of symptomatic adenomyosis. Previous studies demonstrate long-term improvement in patient symptoms and a short-term decrease in uterine volumes, especially in vascular lesions [52].

This systematic review provides a comprehensive analysis of UAE for the treatment of adenomyosis, focusing on technical aspects, clinical outcomes, and post-procedural complications, assessing the current literature available to date on this topic. The review meticulously searched the major scientific databases using well-defined keywords and MeSH, with a period covered from January 2003 to December 2025, ensuring a thorough examination of the updated literature without temporal bias. The inclusion criteria were stringent, focusing on English-language studies with accessible full texts, involving adult female patients, and clearly citing sources. Studies that combined adenomyosis with fibroids were included only if specific adenomyosis-related outcomes were reported. This rigorous selection process ensured the relevance and reliability of the included studies. Our final search included 22 articles, and the study sample covered a total of 1701 women with adenomyosis.

Uterine artery embolization is a minimally invasive interventional radiological technique, first performed by Ravina et al. in 1995 for the treatment of fibroids, and decreasing the arterial supply to the uterus to control abnormal bleeding symptoms. The embolization techniques varied, with most studies using polyvinyl alcohol (PVA) particles of different diameters, ranging from 100 to 710 µm, and some studies using additional materials such as tris-acryl gelatin microspheres and embospheres. The choice of embolization material and particle size could affect both the efficacy and safety of the procedure. Technically, a complete stasis of flow within the uterine arteries is the final endpoint. Calibrated microparticles (sizes ranging from 100 to 710 µm) were used in all procedures, and in more than half of the studies analyzed, embolization was started with smaller particles with subsequent upsizing [31]. However, currently studies on the efficacy of different embolizing materials or the superiority of one over another are lacking, suggesting the need for standardization in future research. Even if technically UAE procedure is similar for adenomyosis and fibroids, a more aggressive approach to reach complete stasis of flow is the main endpoint for the treatment of adenomyosis; in fact, in adenomyosis, the afferent arterioles are generally smaller than in fibroids and the use of smaller particles allows to first block these vessels, reaching better results. On the other hand, patients have experienced more intense pain after UAE for adenomyosis with this technique, suggesting the need to carry out the procedure under patient-controlled analgesia. Pelvic pain was one of the most commonly encountered adverse events of UAE and it was probably the result of the ischemic alteration after embolization [42]. In all studies analyzed, local anesthesia or conscious sedation was performed during procedure. Pain management is a critical component of post-UAE care. In two different studies conducted in 2013 and 2016 on pain management therapies after UAE, Kim SY et al. investigated the use of dexmedetomidine, demonstrating that its infusion reduced opioid consumption and associated side effects, providing better analgesia and less nausea and vomiting, without significant hemodynamic instability. This suggests that dexmedetomidine could be a valuable adjunct in post-UAE pain management protocols. The use of dexamethasone significantly reduced inflammation, pain, and the incidence of severe nausea and vomiting within the first 24 h post-procedure. This supports the inclusion of dexamethasone as an adjunctive treatment to enhance patient recovery and comfort post-UAE [53, 54].

UAE for adenomyosis significantly improves short-term and long-term symptoms. In our study, symptom improvement was reported in at least 50% of patients across all studies, with an average of 76.4% experiencing significant symptom relief. The primary symptoms addressed were heavy menstrual bleeding, chronic pelvic pain, and bulk-related symptoms, and the majority of patients with therapy-resistant adenomyosis experienced improvement of these symptoms and preservation of the uterus. Follow-up periods ranged from 3 to 88 months, averaging 31 months, confirming that UAE is a good therapy for adenomyosis in the short-, mid-, and long-term period. Interestingly, in some studies, longer follow-up periods often revealed a rebound in symptoms, highlighting the importance of sustained post-procedural monitoring through imaging. Bratby and Walker reported a consistent clinical response with an 80% improvement of heavy menstrual bleeding up to 1 year; on the other hand, a 40% recurrence of abnormal menstrual bleeding was reported in women with adenomyosis after 2 years post-UAE [25]; however, none of these women has chosen hysterectomy. These data underscore the need for long-term follow-up and possibly supplementary treatments to manage persistent symptoms. Hysterectomy was the most common reintervention for recurrence of symptoms, followed by secondary embolization and curettage. Factors influencing reintervention rate included the presence of fibroids alongside adenomyosis and the higher initial thickness of the JZ. Froeling et al. highlighted that patients with predominant uterine leiomyoma and adenomyosis were more likely to require reinterventions, emphasizing the need for personalized treatment plans based on initial patient characteristics [27]. Smeets et al. identified thicker JZ as a predictor for hysterectomy during long-term follow-up, while no relation with the need of hysterectomy or clinical outcome was found in patient with the coexistence of fibroids [39].

Morphological changes such as uterine volume and junctional zone thickness reduction after UAE were consistently observed across all studies that evaluated it, with an average reduction in uterine volume of 36.5% and in JZ thickness of 37.2%. Most uterine volume reductions have been confirmed by MRI within 3 to 12 months post-procedure, suggesting that the effectiveness of UAE in reducing uterine volume might be time-dependent. This aspect is crucial for clinicians in setting realistic expectations and planning follow-up care for patients. Uterine volume reduction was also related with relief of bulky symptoms. However, despite some authors hypothesized that the reduction in uterine volume in patients with fibroids could be related to a reduction in the volume of the fibroids themselves, others have demonstrated that there was no difference in uterus volume between the patients with pure AD or AD combined with fibroids and that improvement of symptoms after UAE was not related to the presence of fibroids [45]. Increased endometrial cavity and JZ thickness also contributes to abnormal menstrual bleeding because the higher vessel density associated with adenomyosis; furthermore, total uterine size may worsen bulky symptoms and pelvic pain. Zhou et al. indicated the high grade of vascularity in adenomyosis as a factor which could be predict a better response to UAE [46]. On the same direction, Hu et al. analyzed the improvement of dysmenorrhea and menorrhagia in patients with the absence of contrast enhancement on T1-weighted images of the uterus in postoperative MRI, suggesting the necrosis of adenomyotic tissue as a predictor for mid-term prognosis and for the absence of recurrence at long-term follow-up [23–30]. Therefore, morphological changes after UAE may explain, in part, alleviation of symptoms. Our data on change in uterine volume and JZ falls within the range reported in the literature [32, 41].

Permanent amenorrhea occurred in 2.6% of patients across 22 studies, and all those patients were > 40 years. This complication might result from ovarian ischemia due to non-targeted embolization through utero-ovarian anastomoses. Thus, ovary function and the study of utero-ovarian anastomoses on DSA must be investigated before embolization [55].

In recent years, minimally invasive interventional radiology techniques to treat symptomatic AD have gained attraction as a uterine-sparing therapy with low hospital stay and complication rate. To date, our work confirms previous studies analyzing most recent large-scale studies on UAE of adenomyosis [56, 57].

## Limitations and future perspectives

Our systematic review presents several limitations. The analysis of the articles included in our study was mainly based on retrospective studies, making clinical and statistic data inhomogeneous, and subjecting to bias common to all retrospective studies. Furthermore, randomized controlled trials (RCT) on this topic are still lacking, making it difficult to demonstrate the effectiveness of the treatment. The only “Quality of Life after Embolization vs Hysterectomy in Adenomyosis” (QUESTA) multicenter randomized controlled trial was converted into a prospective cohort study [40]. These aspects represent an important limit in terms of the scientific evidence. Different studies analyzed cohort of patients with both adenomyosis and fibrosis, making it difficult to distinguish whether improvement of symptoms after UAE was related to the treatment of adenomyosis, fibroids, or both. Regarding symptom control, the criteria for improvement of heavy menstrual bleeding and/or dysmenorrhea relied mainly on subjective patient assessment, with no questionnaire that specifically evaluates adenomyosis. The use of different scales and questionnaires was not standardized among studies and a harmonization or sensitivity analysis of results was difficult to implement. In this perspective, the impact of UAE on fertility and pregnancy rates, the association of adenomyosis and endometriosis, and the correlation between known imaging features with the clinical presentation of adenomyosis need to be studied and developed, representing important diagnostic and prognostic values of the disease. As part of this ongoing research, it will be beneficial to calculate the volume of ischemic myometrium in relation to the total uterine volume. This measurement could provide further insights into the efficacy of UAE and help refine treatment protocols to achieve the best possible outcomes for patients with adenomyosis. These could represent interesting future perspectives and require further studies on this topic.

## Conclusions

This systematic review underscores the effectiveness of UAE as an alternative treatment in managing therapy-resistant adenomyosis, particularly in providing significant symptom relief, substantial morphological changes, and long-term preservation of the uterus. Fertility preservation in patients with adenomyosis also offers an additional chance of becoming pregnant compared to other pharmacological or surgical treatments. Improvements in heavy menstrual bleeding, chronic pelvic pain, and bulk-related symptoms in up to 96% of patients, associated with low complication rate following UAE, contribute to a better quality of life for patients during short- and long-term follow-up. Pain management strategies show considerable promise in enhancing post-procedural outcomes.

Future research should aim to standardize embolization techniques and materials, and exploring long-term outcomes will be crucial in optimizing patient care and management strategies. These findings support the need for personalized treatment plans and suggest that women should have access to consult with interventional radiologists when making any treatment decision for adenomyosis.

## Data Availability

The datasets used and/or analyzed during the current study are available from the corresponding author on reasonable request.

## Abbreviations

UAE: Uterine artery embolization
AUB: Abnormal uterine bleeding
TAS: Transabdominal sonography
TVS: Transvaginal sonography
MRI: Magnetic resonance imaging
POI: Premature ovarian insufficiency
PVA: Polyvinyl alcohol particles
TAGM: Tris-acryl gelatin microspheres
UV: Uterine volume
JZ: Junctional zone
HRQOL: Health-related quality of life questionnaire
SSS: Symptom severity scores
QoF: Quality of Life questionnaire
VAS: Visual Analogue Scale
DSA: Digital subtraction angiography
